# Supplementary material for: Recovering from COVID-19 (ReCOV): Feasibility of an Allied-Health-Led Multidisciplinary Outpatient Rehabilitation Service for People with Long COVID
Source: Int J Environ Res Public Health. 2024 Jul 22;21(7):958. doi: 10.3390/ijerph21070958 (PMC11277266; doi:10.3390/ijerph21070958)
Supplement: Supplementary file 1 [file ijerph-21-00958-s001.zip › ijerph-3089559-supplementary.pdf]

Supplementary 1: Template for Intervention Description and Replication - Telehealth (TIDieR- Telehealth)

Item	Details
1 Brief Name	ReCOV
2 Why	To provide multidisciplinary education and rehabilitation for patients with Long COVID for symptom relief and self-management strategies. This service was provided via telehealth to improve accessibility (geographically and for those with fatigue)
3 What: Materials	Initial assessment comprising survey and outcome measures was completed electronically or via phone to determine patient symptoms and needs. Interpreters were provided for assessment and intervention as needed. All telehealth intervention was via Health Direct video call technology.<br>Materials provided (as indicated): Written exercise programs, Handouts, PowerPoint slides from group education and links to further information.
4 What: Procedures	An allied health led multidisciplinary rehabilitation service.<br>Interventions were personalised based on self-reported symptoms via questionnaire responses. Each discipline provided specialist care within their scope of practice to address needs of the participants. Procedures of this service were originally set up for remote delivery via Telehealth, with links generated for participants to attend 1:1 and group sessions.
5 Who	The ReCOV multidisciplinary team comprised of the following professions and all staff were set up with a telehealth login and registered with the Health Direct video call technology.<br>**Occupational Therapy:** 2x senior Occupational therapists with training in fatigue management and pacing<br>**Physiotherapy:** Senior Physiotherapist with clinical experience in vestibular rehabilitation and falls management.<br>**Exercise Physiology:** Senior Exercise Physiologist with specialist expertise in chronic fatigue management and graded return to exercise programs<br>**Clinical Psychology:** Senior clinician with expertise in management of low mood and anxiety<br>**Neuropsychology:** Senior clinician with expertise in executive dysfunction and working memory.<br>**Music Therapy:** 2x senior music therapists with expertise in breath capacity and breathing techniques for anxiety<br>**Dietician:** Junior Dietician with experience in management of malnutrition and nutrition counselling<br>**Social Work:** senior Social worker with experience in trauma management and counselling<br>**Rehabilitation Physician:** senior clinician in management of complex medical presentations and coordination of care<br>**Allied Health Assistant/ Admin:** junior clinician with experience in managing administration tasks and patient communications

6 How	Service delivery: Primarily delivered via telehealth (videoconferencing), face-to-face appointments offered as needed (e.g. vestibular assessment, supervised exercise). Group education and peer support were in addition to individual sessions to cover main themes around patient symptoms and were also delivered via telehealth.
7 Where	Staff were working from home or at the tertiary hospital in an outpatient setting when videoconferencing.<br>Participants were living independently in the community.
8 When/ How	Sessions were provided over a 12-week period per profession; initial appointments scheduled for 1 hour and reviews from 30mins to 1 hour depending on the staff and clinical indication. The number of appointments was determined by clinical indication.
9 Tailoring	Participants received individualised care based on assessment. Sessions were tailored to meet the needs and progress of individuals.
10 Modification	It was planned that all appointments with health care professionals were delivered within 12 weeks but due to wait listing time for certain professions, participants were subsequently offered 12 weeks per profession.<br><br>Team members identified they had been frequently providing education on certain themes during individual sessions.<br>In March 2023, group education sessions were introduced and participants were encouraged to attend two telehealth groups per week, over a 3-week period for education and self-management strategies related to fatigue, cognition, sleep, return to activity, relaxation and mindfulness and breathlessness. Participants then had the opportunity to seek up to 3 individual sessions to address specific needs.
12 How well: Actual	Referrals, wait times and attendance were recorded.<br>Clinical supervision was provided to all clinicians as per standard hospital protocol.<br>Multidisciplinary and Team meetings were held monthly to check service being provided as planned and discuss ongoing patient care.
